# Challenges in the Role of Gammaglobulin Replacement Therapy and Vaccination Strategies for Hematological Malignancy

**DOI:** 10.3389/fimmu.2016.00317

**Published:** 2016-08-22

**Authors:** Silvia Sánchez-Ramón, Fatima Dhalla, Helen Chapel

**Affiliations:** ^1^Department of Clinical Immunology and IdISSC, Hospital Clínico San Carlos, Madrid, Spain; ^2^Department of Microbiology I, Complutense University School of Medicine, Madrid, Spain; ^3^Nuffield Department of Medicine, University of Oxford, Oxford, UK; ^4^Department of Clinical Immunology, John Radcliffe Hospital, Headington, Oxford, UK

**Keywords:** antibody production defect, hypogammaglobulinemia, chronic lymphocytic leukemia, hematological malignancy, multiple myeloma, replacement immunoglobulins

## Abstract

Patients with chronic lymphocytic leukemia (CLL) and multiple myeloma (MM) are prone to present with antibody production deficits associated with recurrent or severe bacterial infections that might benefit from human immunoglobulin (Ig) (IVIg/SCIg) replacement therapy. However, the original IVIg trial data were done before modern therapies were available, and the current indications do not take into account the shift in the immune situation of current treatment combinations and changes in the spectrum of infections. Besides, patients affected by other B cell malignancies present with similar immunodeficiency and manifestations while they are not covered by the current IVIg indications. A potential beneficial strategy could be to vaccinate patients at monoclonal B lymphocytosis and monoclonal gammopathy of undetermined significance stages (for CLL and MM, respectively) or at B-cell malignancy diagnosis, when better antibody responses are attained. We have to re-emphasize the need for assessing and monitoring specific antibody responses; these are warranted to select adequately those patients for whom early intervention with prophylactic anti-infective therapy and/or IVIg is preferred. This review provides an overview of the current scenario, with a focus on prevention of infection in patients with hematological malignancies and the role of Ig replacement therapy.

## Introduction

Patients affected by hematological malignancy, in particular chronic lymphocytic leukemia (CLL) and multiple myeloma (MM), were recognized as presenting antibody production deficits many years ago (1, 2), and the proposal was made that they might benefit from antibody replacement therapy (3). Landmark clinical trials in CLL and MM in the late 1980s and early 1990s settled the basis for the current indications of intravenous immunoglobulins (IVIg) in hematological malignancies associated with severe secondary hypogammaglobulinemia and recurrent infections (4–6). In recent years, the convergence of better immunological evaluation of antibody responses for the selection of patients who might benefit from immunoglobulin replacement therapy, together with substantially improved therapies for these malignant conditions that lengthen survival, has prompted the need to review the role of IVIg (or subcutaneous administration, SCIg) in the prevention of infectious complications. Besides, there are other B cell malignancies with similar immune defects that are not currently authorized as indications for IVIg, thus resulting in subsequent discrimination of such patients.

### Outline of Article

First, we briefly review the current evidence for the immunological approach to the prevention of infectious complications associated with defective antibody responses in CLL and MM. We then survey the current challenges derived from the improvements in diagnosis and therapy of hematological malignancies and discuss some practical issues. Finally, we consider the potential indices for better selection of patients most likely to benefit from early intervention with IVIg or SCIg.

## Current Evidence-Based Guidelines for Immunoglobulin Therapy in Hematological Malignancies

Recurrent or severe infections are a major cause of morbidity and mortality in patients with CLL (between 30 and 50% of deaths) (7–9) and MM (22% of deaths in the first year after diagnosis) (10), especially in patients with renal failure (11).

Hypogammaglobulinemia (low serum levels of IgG and IgA with variable IgM) is a well-recognized complication associated with hematological malignancy, most commonly in CLL and MM, while not common at diagnosis and during the natural history of other B cell malignancies (12). It is present in about a 25% of patients at diagnosis and up to 85% during the disease course, rendering them susceptible to infections (7, 9, 13, 14). It occurs in patients with mutated and unmutated immunoglobulin heavy chain (IGHV) genes (7). Nevertheless, other immune defects are present in these patients mostly due to the underlying disease and to chemotherapeutic protocols, namely neutropenia, mucosal lesions, T-lymphocyte dysregulation and altered cytokine secretion profiles, complement activation (15–17), NK cell dysfunction (18), phagocytic alterations (19, 20), as well as the elderly age of such patients and their poor functional status (7, 21–23).

Complex causes of these low immunoglobulin levels (Igs) include defective production of polyclonal Igs due to abnormal function of non-clonal CD5^−^ B cells; impaired IgG and IgA class-switch through abnormal CD40–CD40L interaction and down-modulation of CD40L (24, 25); impaired help and excessive suppression by T-cells (26, 27); sequestration of T-cell help by CLL cells in pseudofolicles (7), inhibition of CD95^+^ plasma cells in the bone marrow *via* interaction with CD95L on CLL B-cells (28), and iatrogenic myelosuppressive chemotherapy (9, 21).

Data from six randomized clinical trials in CLL and one with MM patients with hypogammaglobulinemia and history of infections demonstrated that IVIg significantly decreased the rate of bacterial infections and prolonged the time to first infection, with no differences in non-bacterial infections (Table 1). These trials suggested that the best dosing was 400 mg/kg/3 weeks until steady state is reached, followed by 400 mg/kg/5 weeks (grade A recommendation, level 1b evidence) (4–6, 29–33). Although infections are a major cause of morbidity and mortality in CLL, neither survival benefit nor improvement in quality of life could be demonstrated, which is not surprising given the follow-up period of 1 year (4, 34). A recent 14-year retrospective study in a large series of CLL patients confirmed that hypogammaglobulinemia does not appear to impact overall survival (14). Based on the results of the first controlled trial in a wide range of CLL patients, IVIg was not cost-effective (35). In patients with MM, IVIg for 6–12 months reduced the risk of severe infectious complications (grade A recommendation, level 1b evidence) (31). As a result, IVIg is currently reserved for selected CLL patients with hypogammaglobulinemia and recurrent bacterial infections, especially those in whom prophylactic antibiotics have failed, or with severe infections requiring IV antibiotics or hospitalization and serum IgG levels <400 mg/dL (grade 2B recommendation, level 1A of evidence). Following the original trial, IVIg may be recommended for plateau phase MM patients with hypogammaglobulinemia and recurrent bacterial infections who have failed to respond to pneumococcal immunization (36, 37).

**Table 1:** **Clinical trials to determine effectiveness and dosage of replacement intravenous immunoglobulin in hematological malignancy [adapted from Dhalla et al. (9)]**.

Publication	Target population	Study description	Relevant results
Cooperative Group (4)	CLL patients (81) with hypogammaglobulinemia or serious infections	Multicenter controlled, randomized double-blind, IVIg 400 mg/kg/21 days versus placebo for 12 months	Fewer major and moderate bacterial infections overall
			Longer period to first serious bacterial infection
			No differences in viral and fungal infections
Griffiths et al. (29)	CLL (8) and low grade NHL (4) patients with hypogammaglobulinemia or serious infections	Double-blind, randomized crossover IVIg 400 mg/kg/21 days versus placebo for 12 months then changed to the alternative drug	Fewer major and moderate bacterial infections overall
			Serious bacterial infection showed a growing trend with IgG < 6.4 g/L
			No differences in trivial infections
Chapel et al. (31)	MM patients (83) with hypogammaglobulinemia or infections	Double-blind, randomized IVIg 400 mg/kg/21 days versus placebo	Fewer life-threatening and severe and recurrent infections
			Maximum benefit in patients with poor pneumococcal response
Chapel et al. (5)	CLL patients (34) with hypogammaglobulinemia and infections	Double-blind, randomized IVIg at either 500 or 250 mg/kg/28 days	Similar rates of infection
Jurlander et al. (32)	CLL patients (15) with hypogammaglobulinemia and recurrent infections	Open label IVIg 1,000 mg/21 days	Fewer hospital admissions and febrile episodes
			No difference in severe infections
			No difference in antibiotic prescription
Sklenar et al. (30)	CLL (31) and MM (31) patients	Multicentre double-blind, randomized parallel-group IVIg at 100, 400, and 800 mg/kg/21 days	Optimal dose was 400 mg/kg for prevention of bacterial infections and for increasing pneumococcal antibody levels
Boughton et al. (6)	CLL patients (42) with hypogammaglobulinemia and infections	Randomized parallel-group IVIg 18 g/21 days versus placebo and switched to 24 g versus 18 g if ≥3 infections	Fewer serious and moderate bacterial infections
			50% who required dose increase subsequently infection free
			Majority of infections associated with IgG < 3 g/L

*CLL, chronic lymphocytic leukemia; MM, multiple myeloma; NHL, non-Hodgkin lymphoma*.

However, most of the trial data on which these recommendations are based is over 20 years old. The spectrum of infections has changed in the last decade. Encapsulated bacteria (*Streptococcus pneumoniae, Staphylococcus aureus*, and *Haemophilus influenzae*) and herpesviruses remain the most prevalent cause of infections in CLL patients, mainly of the respiratory tract but also the skin, urinary and gastrointestinal tracts, and bloodstream (9, 38, 39). Additional pathogens of concern vary depending upon the treatment regimen (discussed below).

The original IVIg trial data were done before modern therapies were available. The current guidelines do not take into account the shift in the immune situation of current treatment combinations (40). On the other hand, the above treatment studies were performed without specific antibody testing for patient selection. The use of similar chemotherapeutical protocols in other lymphoproliferative syndromes, mainly indolent non-Hodgkin lymphomas (iNHL) (41, 42), suggests that there may be low serum Igs in these patients not previously included in trials (43). This leaves a number of patients without access to IVIg. Regarding MM, although a profound antibody deficit may occur before the plateau phase, and the risk of fungal and viral infections is highest during the first 3 months after diagnosis and therapy, there have been no new trials of infection prevention with IVIg (37). In view of the above, there is a need to re-assess both the level of antibody failure and the role of IVIg on the basis of earlier diagnosis and newer treatment modalities.

## Specific Antibody Production Responses in CLL/MM: Immunological Evaluation and Monitoring

### Specific Antibody Responses in B-Cell Malignancies

Not all patients with hypogammaglobulinemia present with infectious complications, so a poor response to pneumococcus was suggested as a good predictor of infections (13, 31); furthermore, serious infections can occur in the absence of hypogammaglobulinemia due to other reasons such as neutropenia or T cell suppression. Low baseline levels of specific exposure antibodies against various bacterial, viral, fungal, and protozoan pathogens have been described in these disorders (44). B-cell antimicrobial dysfunction increases progressively from monoclonal gammopathy of undetermined significance (MGUS) to Waldenstrom macroglobulinemia to MM (31, 44). In therapy-naive CLL patients, seroconversion from positive to negative IgG values was noted for EBV- (3.6%) and, most frequently, for VZV-specific IgG (18%), while IgG specific for CMV was preserved (45). Significantly, lower responses to vaccination in CLL patients with respect to healthy subjects have been reported against diverse antigens. Specific antibody production to polysaccharide antigens (T-cell-independent response), such as classical 23-valent pneumococcal vaccine (PPV23), is markedly impaired in these patients (13, 31, 46). Defective response to protein antigens, such as tetanus toxoid and influenza virus, is also apparent (47, 48). Interpretation of vaccine responses is complex and requires diverse clinical considerations (49). For the evaluation of primary responses, an increase greater than threefold after 4 weeks with respect to prevaccination levels is considered normal (49). The use of *Salmonella typhi* Vi vaccine (50) with pure polysaccharide extract may add clinical value in this population.

### Immunological Evaluation in B-Cell Malignancy

To evaluate the role of immunological deficiencies and to monitor patients with hematological malignancy, a complete clinical history of infections is recommended at diagnosis and during follow-up, as well as quantification of serum immunoglobulins (51) and circulating lymphocyte subsets, including CD4 and CD8 T cells as well as B cells (provided the B cell count in CLL is not excessively high) (Table 2). Neutrophil counts should be also regularly monitored.

**Table 2:** **Initial proposed immunological evaluation in patients with hematological malignancy**.

**Mandatory**
Detailed medical history. History of recurrent or unusual infections, family history
Complete physical examination, including the skin, all mucous membranes, lymph nodes, spleen, and rectum
CBC with manual differential (presence of anemia, neutropenia, lymphopenia, and thrombocytopenia)
Quantitative IgG, IgA, IgM, and IgE levels
**Highly recommended tests**
Isohemagglutinin titers
IgG antibody titers to prior immunizations/exposure
Antibody response to vaccine antigens (e.g., non-conjugated and conjugated pneumococcal, tetanus, diphtheria, *S. typhi*, meningococcal antigens, *Haemophilus influenzae* b)
T and B cell subsets immunophenotyping and absolute counts
**Additional tests**
Lung function tests
Thoracic CT
Memory B cell phenotype
Autoantibodies in autoimmune phenomena: antinuclear, anti-DNA, antiphospholipid, anti-platelet and anti-neutrophil antibodies, cold agglutinins

A recent review by Dhalla et al. (9) has highlighted the relevant role of routine immunological evaluation for secondary specific antibody deficiency to protein and polysaccharide immunizations in CLL as a method for predicting patients prone to infections. These responses should be monitored every 6–12 months and after significant bacterial infections or immunosuppressive therapy, and this approach could be extended to other hematological malignancies.

IgG subclass evaluation could be useful. In a large series of CLL patients, subclass deficiency (particularly IgG3 and IgG1 subclass deficiency) better correlated with recurrent or significant infections than hypogammaglobulinemia itself (100% of IgG subclass deficiency versus 50% of hypogammaglobulinemia, respectively) (52). In another study, decreased concentrations of IgG4 and IgG2 were associated with increased susceptibility to infection (17). However, other studies have not shown association between IgG subclass deficiency and infection in CLL (53).

A recent study showed more serious infections in secondary than in primary antibody deficiency patients and similar diagnostic delay and incidence of bronchiectasis (54). For early detection of preventable lung involvement, pulmonary function tests and high-resolution computerized lung tomography are essential to prevent development and/or progression of bronchiectasis (9). Our strong recommendation is to always consult a clinical immunologist for performing immunological evaluation.

## Diagnosis and Therapy Issues Challenging the Role of Prevention with Intravenous/Subcutaneous Gammaglobulins

Authorized indications may not be aligned with the current clinical scenario, which stems from diagnostic and therapy changes in hematological malignancies in recent years.

The 2008 revised WHO Classification of Tumours of Haematopoietic and Lymphoid Tissues (55) adopted consensus guidelines for the definition of some well-established diseases, including CLL and MM under the common denomination of mature B-cell neoplasms with other entities. According to this classification, CLL and small lymphocytic lymphoma (SLL) are recognized as different manifestations of a single disease entity (56). Some patients may present solely with lymphadenopathy, organomegaly, and presence of infiltrating monoclonal B cells with the same immunophenotype as CLL cells, but lacking peripheral blood lymphocytosis (57). Moreover, CLL patients are currently being diagnosed earlier in life than was previously the case and the distinction of monoclonal B lymphocytosis (MBL) from CLL is based on practice guidelines of 5,000 lymphocyte counts/μL, as no proven biological parameter can distinguish MBL from CLL or identify which patients will progress to clinically significant disease more rapidly (58, 59). It appears that the prevalence of cases that present as “SLL” may be much lower than that of CLL (60). CLL is classified separately as SLL in the International Classification of Diseases (ICD)-10-CM 2015 system (ICD-10).

In the revised 2008 classification, there were no recommendations for changing the definition of recognized categories of MGUS, and regrouped the continuum of smoldering myeloma and indolent myeloma in asymptomatic myeloma. The presence of radiographically detected bone lesions, even if not symptomatic, would exclude a patient from this category, because these are an indication for treatment (55, 56). To widen the scope of IVIg use and to favor access for other B-cell malignant neoplasms, the Canadian guidelines of IVIg use in secondary immunodeficiencies established the indication as infection prophylaxis in adults with “malignant hematological disorders” associated with secondary hypogammaglobulinemia and either a recent life-threatening infection, which is thought to be caused by low levels of polyclonal Ig or recurrent episodes of clinically significant infections necessitating the use of antibiotics and which are reasonably thought to be caused by low levels of polyclonal immunoglobulins (23).

### Emerging Infections with Novel Therapies

Current front-line treatment strategies for CLL including combination chemotherapy and biologic agents, such as fludarabine plus cyclophosphamide plus Rtx (FCR) or bendamustine plus Rtx (BR) or alemtuzumab, have greatly improved overall survival, complete response rate and progression-free survival (61–65). However, it has been suggested that these combination regimens increase synergistically myelosuppression and immunosuppression, which comes to the prize of an increased risk of infections (66, 67). Severe or unusual infections, with higher rates of global infections compared with the historical group of patients treated with FC alone but without significant influence in infection-related mortality have been reported (62). Particularly, the growing use of Rtx in front-line combination chemotherapy with fludarabine (FCR) increases the risk of all types of infections with respect to Rtx in combination with cyclophosphamide, doxorubicin, vincristine, and prednisone (R-CHOP) or with cyclophosphamide, vincristine, and prednisone (R-CVP) chemotherapy (68). FCR regimen results in a significant myelosuppression and high rates of early and late infections, especially in elderly patients (61). With respect to maintenance therapy, Rtx alone for 2 years after induction therapy was associated with higher rate of all types of infections than the observation alone group (66 versus 50%) without influence in infection-related mortality (69). Anti-CD20 monoclonal antibodies have been associated with CMV infections and other virus reactivations (hepatitis B and polyomavirus JC) (63, 70, 71). Reactivation of herpes viruses have also been described related to treatment with purine analogs (as Flu) and alemtuzumab (8). With FR combination but also alemtuzumab, opportunistic infections by *Pneumocystis, Listeria*, mycobacteria, and *Candida* have become a real concern and occur frequently during the first 3 months of therapy and in previously treated patients (7, 72–74). Second- and third-generation anti-CD20 monoclonal antibodies are humanized or fully human antibodies used in CLL and indolent NHL with either higher complement-dependent cytotoxicity or antibody-dependent cell-mediated cytotoxicity or direct cytotoxic effects than Rtx (75), but their infections profile in the long-term respect to that of Rtx remain to be explored. Obinutuzumab use for hematological malignancy was associated with severe infection in 7% of patients in a recent clinical trial (76).

The impact of new biologicals for hematological malignancies that inhibit B-cell receptor (BCR) signaling pathway, B cell leukemia/lymphoma-2 (Bcl-2) antagonists, and chimeric antigen receptor (CAR) T cells in terms of long-term infectious complications has not been established. BCR inhibitors’ approved to date include the Bruton’s tyrosine kinase (BTK) inhibitor ibrutinib; the selective inhibitor of the phosphatidylinositol 3-kinase (PI3K) delta isoform idelalisib; as well as Syk inhibitor fostamatinib disodium (FosD) and SRC family kinases inhibitors (dasatynib, bafetinib). A recent study reports ibrutinib to have a better impact on humoral reconstitution and hence lower infection rates at 12 months (77). However, in a large multicenter, open-label, clinical trial evaluating ibrutinib versus anti-CD20 (ofatumumab) therapy in refractory CLL, severe infections (mainly pneumonia, urinary tract infections, and pyrexia) were similar in both groups (24 versus 22% of patients), with higher infections in general in the ibrutinib group (70 versus 54%, respectively), although patients on ibrutinib had longer drug exposure (median, 8.6 versus 5.3 months) and more than 3 months longer adverse event reporting period (78). The rate of severe infections was lower when ibrutinib was given as first-line therapy (4% pneumonia and 4% diarrhea) (79). In the clinical trial with idelalisib for refractory CLL reported infections included pneumonia in 6% and febrile neutropenia in 5% (80); another clinical trial with idelalisib for indolent refractory NHL included severe neutropenia in 27% and pneumonia in 7% (81), while a clinical trial on treatment-naive CLL patients reported 18% incidence of pneumonia (82). Bcl-2 antagonists, such as venetoclax, have shown in a recent phase II clinical trial in refractory CLL an incidence of pneumonia in 6% and febrile neutropenia in 5% of patients (83). In summary, despite promising clinical efficacy and acceptable adverse effects’ profiles, these later agents inhibit key molecules in B cell activation and differentiation and also other relevant pathways independent of BCR and neutrophils. Thus, the real clinical extent of the humoral and cellular immune and innate defect of these new agents at the long term, as shown by the primary defects of these molecules (84, 85) remains to be determined.

On the other hand, CD19 CAR T cells have been used in relapsed and high-risk CLL with overall response rate of 57% of 14 patients and persistent B cell aplasia in 100% of patients with complete remission (CR) and half of the patients with partial remission (86, 87). One out of the 4 patients who achieved CR died of an infectious surgery complication almost 2 years after CAR T therapy. Severe hypogammaglobulinemia in these patients was managed with IVIg (86). Interestingly, long-lived plasma cells may account for the persistence of secondary responses to previous exposures (88).

Similar front-line treatment regimens as for CLL (mostly FluR combination) and novel biologic agents are being used in iNHL, mostly in the management of follicular lymphoma (FL), mantle-cell lymphoma (MCL), and cases of diffuse large cell lymphoma (DLCL), leading to substantial improvement in prognosis and overall survival (89–92).

The issue of immune hyporesponsiveness during specific intensive chemotherapy is still unexplored. Secondary antibody responses do not seem to be invariably impaired after anti-CD20 monoclonal antibodies, but primary responses, as tested in numerous immunization studies, are clearly impaired (93, 94). Rtx has been associated with prolonged hypogammaglobulinemia (95, 96) and with neutropenia (even with delayed-onset) (97), although difficult to quantify due to confounding factors, namely, concomitant use of these biologicals with other immunosuppressive or chemotherapeutic agents and the underlying conditions, as well as under-reporting (98). The experience learned from Rtx for autoimmune disease demonstrates that the lower the baseline IgM and IgG levels, the lower post-treatment IgM and IgG levels, the higher infection risk (96, 99), with an accumulative effect after repeated cycles (100).

## Practical Issues in the Prevention of Infectious Complications

### Vaccination

Protecting immunocompromised patients against vaccine-preventable infectious diseases is a commonly missed opportunity (101). Although there are no randomized studies showing that vaccination may alter infection rates or outcomes from acquired infections in CLL, routine vaccinations should be maintained in these patients before initiation of treatment if possible (39, 102). All live vaccines are contraindicated, including zoster, since severe and even fatal complications have been reported. Diverse studies have shown reasonable rates of seroprotection and seroconversion in various immunocompromised hosts, including oncology patients, with very minimal downside (101). Conjugate vaccines have proved to be highly immunogenic and are to be recommended in these patients (46, 103).

Therefore, subunit vaccines against seasonal influenza and against H1N1 are broadly recommended across oncologic patient populations, given the severity of the H1N1 pandemic and the highly severe flu impact respect to general population despite poor responses in CLL, and even two doses regimen (48, 101, 104–107).

*Streptococcus pneumoniae* is the commonest pathogen in hematological malignancy, and invasive disease is significantly higher in MM and more modestly in CLL and NHL. As discussed earlier, although vaccination of seven-valent pneumococcal vaccine show protection to six of the seven antigens in 40% in series of CLL patients when administered early and prior to therapy (108), there is no data on infection prevention by such immunization in CLL. Despite this, it is now recommended to administer both the 13-valent and the 23-valent vaccine (107, 109), since 58% of untreated CLL patients showed a response (103).

A moderate vaccination response rate of 43% against *H. influenzae* type b (Hib) conjugate vaccine among adult and elderly patients with CLL has been reported (110) but will only protect against type b *H. influenzae* and not against the much more common infection by the non-encapsulated pathogen. Normal IgG1 and IgA concentrations were associated with protection, while IgM, in turn, was the best predictor of a significant vaccination response.

Patients receiving intensive chemo- or immunotherapy should be screened for hepatitis B and C infection (grade of evidence A1). Hepatitis B vaccine is indicated for patients with CLL who are negative for antibodies against HBsAg; the combined hepatitis A and B vaccine should be considered (111) in endemic regions. The HPV vaccine may be considered for all HPV-seropositive subjects with hematological malignancies, as these malignancies have been associated with high risk of second primary malignancies in general (112, 113), and with HPV in particular in a recent study (not only skin cancer but also prostate and colon cancer cells were HPV DNA by PCR) (114).

Given the suboptimal immune responses to immunization of immunocompromised patients, the optimal timing dosing, use of adjuvants, and delivery method might maximize the immunologic benefit of vaccination in oncology patients (101). A potential beneficial strategy could be to vaccinate patients early at diagnosis, for instance at MBL stage, when better responses are attained than CLL (115). The same might be true for MGUS patients at risk of developing MM.

### Prophylactic Antibiotics

The changing spectrum of infections in CLL patients on current therapeutic protocols mandates a newer approach to prophylaxis and therapy. Early recognition of patients susceptible to infection and prophylactic administration of appropriate antibiotics remain the first-line management for symptomatic antibody deficiency in CLL (9, 21, 22).

However, clinical trials are lacking (51). In patients with bronchiectasis, nebulized or low dose oral antibiotics, such as azithromycin, can reduce the incidence of recurrent infection. Replacement Ig should be considered if, despite AB prophylaxis, there are significant breakthrough bacterial infections or if bronchiectasis develops or progresses (51).

Anti-infective prophylaxis is recommended for patients receiving purine-analog and/or alemtuzumab during treatment and thereafter, if tolerated herpes virus (acyclovir or equivalent) and anti-*Pneumocystis jirovecii* prophylaxis (sulfamethoxazole/trimethoprim or equivalent) (Grade of evidence IV, B) (39, 51, 107, 116). Antivirals are given if the patient is on chemotherapy (FC or FCR) and thus at high risk of opportunistic viral infection. Anti HSV and HZV prophylaxis is recommended in patients requiring intensive and/or immunosuppressive treatment who are seropositive, have a low CD4 count or a history of previous herpes infections (51). The duration of anti-*Pneumocystis* and herpes prophylaxis is controversial.

Patients on alemtuzumab should be monitored for CMV reactivation (117–119). The current management is controversial; some use ganciclovir (oral or IV) prophylactically if viremia is present, others use ganciclovir only if viral load is rising. CMV viremia should be measured by PCR quantitation at least every 2–3 weeks. Pre-emptive ganciclovir/valganciclovir may be used with CMV viremia or increasing viral load, for 14–21 days until symptoms resolve and PCR tests are negative. Patients’ positive for HBSAg or HBCAg may require antiviral treatment and should be managed jointly with a specialist in viral hepatitis (120).

### Growth Factors

The myeloid growth factor, granulocyte colony-stimulating factor (G-CSF) or granulocyte macrophage colony-stimulating factor (GM-CSF), has radically changed the approach to the prevention of febrile neutropenia (121–123). Their use is recommended for the first cycle of chemotherapy for patients with more than a 20% risk of febrile neutropenia or in case of poor response to antibiotics with persistent neutropenia should lead to the use of these cytokines.

### Role of Prophylactic Replacement Immunoglobulin

Given that therapy of hematological malignancies has substantially changed in the last decade with the introduction of drugs with different modes of action, including monoclonal antibodies, drugs with immunomodulatory effects such as lenalidomide, and targeted drugs such as tyrosine kinase inhibitors, the previous clinical trials may not accurately represent the drawbacks and benefits of IVIg/SCIg now.

Current guidelines state the use of IVIg in severe hypogammaglobulinemia and recurrent infections (Level of evidence I, A) (107). The question of the correct dose of IVIg to prevent bacterial infections is still unresolved and is probably patient dependent (9, 37, 124). Much has been learnt from the management of patients with PID, such as the use of clinical measures and trough IgG levels to guide dose adjustments and the need to use higher replacement doses in patients with bronchiectasis (124). As a general guideline, maintenance of trough serum IgG in treated patients above 500–700 mg/dL is a reasonable goal (9).

Treatment should be reconsidered if there is no improvement in the frequency or severity of bacterial infections after 1 year (125). The choices of subcutaneous Ig and self-infusion have reduced costs and improved quality of life (9, 126).

The use of IVIg prophylaxis in hematopoietic stem cell transplantation (HSCT) for MM patients without selection by immunological parameters has shown beneficial results in terms of quality of life, but does not seem to affect CMV infection incidence or other infectious complications (127–129).

Expected mechanisms of action of replacement Ig therapy on B cell cancer include effective reduction in infections by pathogen neutralization, toxin inactivation and opsonization, and complement-mediated bactericidal effects (130). The potential immunomodulatory effects of Ig therapy on the setting of cancer are unclear (131). Ig could induce *in vivo* beneficial immunomodulatory effects on the malignant B cell clone by several potential mechanisms: inducing unresponsive anergy and subsequent apoptosis in B cells, by inducing proapoptotic molecules expression on tumor cells or by Fas-blocking antibodies in IVIg (132) or through ERK phosphorilation *in vitro* (133); altering activation and proliferation of B cells (132, 134); inhibition of B cell antigen presentation (135, 136); *in vitro* differentiation of B cells and Ig secretion (137); and other immune effects [revised by Corbi et al. (131)].

Increases in the immunodeficiency status of CLL and other hematological malignancies, and growing antibiotic resistant pathogens in what is being called the post-antibiotic era, have renewed attention in antibody therapy. In carefully selected patients by clinical infectious history and antibody production defect (9), the criteria for initiating IVIg therapy need to be reassessed in the light of data of PID management to prevent lung damage due to ongoing subclinical infection (138, 139). In PID, replacement IVIg therapy is the mainstay of treatment of subjects with antibody failure, and it has revolutionized the lives of these patients (139).

Futures studies should address the question whether in CLL patients requiring long-standing anti-infectious combined protocols with antiviral, antibiotic, and antifungal treatment, IVIg would add a benefit, given the occurrence of pathogens’ antibiotic resistance, deleterious effects on microbiota and side-effects. Also, comparison studies of the impact of IVIg versus antibiotics with current protocols in terms of lung involvement and prognosis are necessary. In the absence of specific studies, recommendations for IVIg replacement in hematological malignancies should be based on clinical experience in PIDs and only in selected individual patients.

## Potential Indices to Better Select Patients for Whom Early Intervention with IVIg may be Beneficial

Chronic lymphocytic leukemia is a heterogeneous disease that may evolve as indolent or as an aggressive malignancy. There is a still a lack of biological markers underlying different clinical presentations (57).

We propose the following aspects to be considered in order to improve the rational use of IVIg in indication of B-cell malignancy:

1. Selection of patients for Ig replacement therapy by proven antibody production deficit by routine to protein and polysaccharide immunization protocols: antibody production deficit is a better tool than hypogammaglobulinemia to measure functional dysregulation and to define those patients with hematological malignancy that would benefit from IVIg replacement therapy.
2. Besides, timely *Ig* replacement therapy can prevent structural lung damage and progression.
3. Immunological monitoring is important to detect patients at risk of severe infections or development or progression of lung damage.
4. On the basis of the currently available indications, the question arises whether we should consider widening the IVIg indication for “hematological malignancy” with recurrent or severe infections and antibody production defect to other B cell malignancies. This measure may avoid discrimination of patients with of SLL and iNHL with secondary antibody deficiency to CLL and similar chemotherapeutic protocols (140).
5. It would be interesting to ascertain whether immunophenotypic and cytogenetic biomarkers – 17p deletion, ZAP and CD38 expression, and unmutated IgH-, considered as prognostic predictors in terms of overall survival, are associated with more profound immunodeficiency.
6. Assessment of the long-range effects in terms of infectious complications and of immunosuppression of novel agents (i.e., BCR signaling pathway inhibitors and BCL2 antagonists) in combined regimens for indifferent indications (first-line versus repeated cycles) and of CAR T cell therapy.

## Conclusion

As better combination regimens with monoclonal antibodies and new biological agents and cell therapies are being developed, a profound immune dysregulation in patients with CLL and other hematological malignancies occurs, which clearly impact on the clinical course of the disease. A potential beneficial strategy could be to vaccinate patients at MBL and MGUS stages (for CLL and MM, respectively) or at the time of B-cell malignancy diagnosis, when better antibody responses are attained. We have to re-emphasize the need for evaluating and monitoring antibody responses to adequately select patients in whom early intervention with prophylactic anti-infective therapy and IVIg is warranted. New clinical trials would be necessary to establish the role of IVIg in hematological malignancy during the combination front-line protocols against antibiotic prophylaxis and to re-evaluate the cost-effectiveness of IVIg in this new scenario.

## Author Contributions

SS-R, FD, and HC participated in the conception of the work. SS-R wrote the first draft. FD and HC critical revised and wrote the manuscript.

## Conflict of Interest Statement

The authors declare that the research was conducted in the absence of any commercial or financial relationships that could be construed as a potential conflict of interest.
